# The use of acupuncture in patients with Raynaud’s syndrome: a systematic review and meta-analysis of randomized controlled trials

**DOI:** 10.1177/09645284221076504

**Published:** 2022-05-24

**Authors:** Fangwen Zhou, Emma Huang, Elena Zheng, Jiawen Deng

**Affiliations:** 1 Faculty of Health Sciences, McMaster University, Hamilton, ON, Canada; 2 Faculty of Applied Health Sciences, University of Waterloo, Waterloo, ON, Canada

**Keywords:** acupuncture, meta-analysis, network meta-analysis, Raynaud’s phenomenon, Raynaud’s syndrome

## Abstract

**Objective:**

To assess the effectiveness of acupuncture for the treatment of Raynaud’s syndrome by conducting a systematic review and meta-analysis of randomized controlled trials (RCTs).

**Methods:**

Studies were identified from English and Chinese databases from their inception to September 2020. The outcomes of interest were remission incidence, number of daily attacks, incidence of positive cold stimulation tests and incidence of cold provocation tests. We conducted meta-analysis and network meta-analysis using meta and gemtc.

**Results:**

Six trials (n = 272 participants) were included in the meta-analysis. Pairwise meta-analyses show that acupuncture was associated with increased remission incidence (risk ratio (RR) = 1.21, 95% confidence interval (CI) = 1.10 to 1.34), decreased daily number of attacks (weighted mean difference (WMD) = −0.57, 95% CI = −1.14 to −0.01), and increased incidence of positive cold stimulation tests (RR = 1.64, 95% CI = 1.27 to 2.11). There was not enough evidence to associate acupuncture with decreased incidence of positive cold provocation tests. The network meta-analyses did not demonstrate significant results for the effectiveness of any acupuncture treatments (electroacupuncture or manual acupuncture ± moxibustion), compared with controls, in terms of remission incidence or daily number of attacks, possibly due to small sample sizes and a lack of statistical power.

**Conclusion:**

The use of acupuncture may be effective for the treatment of Raynaud’s syndrome in terms of increasing remission incidence, decreasing daily number of attacks and increasing incidences of positive cold stimulation tests. However, our findings should be interpreted with caution due to small sample sizes, very low quality of evidence and high risk of bias. Future large-scale RCTs are warranted.

## Introduction

Raynaud’s syndrome, a phenomenon characterized by color changes in the digits due to exaggerated vasospasms,^1^ is a prevalent condition that occurs in 3%–5% of the global population.^2^ Its onset is typically triggered by cold temperatures, emotional stress, or other medical and environmental factors.^1^ During a Raynaud’s episode, commonly referred to as an “attack,” the affected digits often undergo a three-phase color change: an initial ischemic phase, when the digits turn white (pallor); a deoxygenation phase, when the digits turn blue (cyanosis); and a reperfusion phase, when the digits turn red (erythema).^1^

Raynaud’s syndrome can be classified clinically as primary or secondary.^3^ Primary Raynaud’s syndrome, the most common type, is idiopathic,^3^ whereas secondary Raynaud’s syndrome is usually caused by conditions such as autoimmune diseases and cancer, as well as lifestyle choices such as smoking and medication use.^4^ Some patients with Raynaud’s syndrome, especially secondary, may experience attacks that are frequent and painful, and can lead to digital ulcerations.^1^ To mitigate these symptoms, patients with Raynaud’s syndrome are usually treated with pharmacotherapies such as calcium channel blockers (which are first-line treatments).^1^ However, these may cause a variety of different adverse reactions, such as vasodilation, gastrointestinal effects, and drug-drug interactions.^5^ Because of these side effects, many patients with Raynaud’s syndrome have turned to complementary and alternative medicine (CAM) to manage their symptoms.^6^

Acupuncture, a practice that originated from China, has recently been identified in the field of CAM as a potential therapeutic procedure with supportive scientific evidence.^7^ Recently, there have been several randomized controlled trials (RCTs) investigating its use in Raynaud’s syndrome; however, these studies have often had low sample sizes and therefore have tended to produce inconclusive results.^8^ One solution to this issue is to conduct a meta-analysis, which enables the pooling of outcome data to increase patient sample size and statistical power, thus allowing for a more precise estimate of the treatment effects of an intervention.^9^ Therefore, we conducted a systematic review and meta-analysis of RCTs with the objective of investigating whether the use of acupuncture would result in increased remission rates and decreased daily numbers of attacks in patients with Raynaud’s syndrome.

## Methods

We conducted this systematic review and meta-analysis in accordance with the Preferred Reporting Items for Systematic Reviews and Meta-Analyses (PRISMA) framework.^10^ The PRISMA checklist for this study is shown in Supplemental Table S1.

### Study identification

We searched the following databases from their inception to 17 September 2020: (1) MEDLINE; (2) EMBASE; (3) Web of Science; (4) Cumulative Index of Nursing and Allied Health Literature (CINAHL); (5) The Cochrane Library; and (6) Scopus.

We also systematically searched the following Chinese databases from their inception to 17 September 2020 using a Chinese search strategy: (1) Wanfang Data; (2) Wanfang Med Online; (3) China National Knowledge Infrastructure (CNKI); (4) Chongqing VIP Information (CQVIP); and (5) SinoMed.

The reference sections of previous reviews identified from database searches and clinical trial registrations published before 17 September 2020 in the following trial registries were also hand searched for relevant trials: (1) ClinicalTrials.gov; (2) World Health Organization (WHO) International Clinical Trials Registry Platform; (3) European Union (EU) Clinical Trial Register; and (4) Chinese Clinical Trial Registry.

The search strategy used for the database searches can be found in Supplemental Table S2/S3.

### Eligibility criteria

In order to be included in our analysis, parallel RCTs needed to have: (1) recruited patients diagnosed with Raynaud’s syndrome; (2) used any acupuncture therapy compared with an untreated or sham acupuncture group; and (3) reported any of our outcomes of interest.

We included studies that may have administered other therapies concurrently with acupuncture; however, the same concurrent therapy needed to have been used in both the intervention and control arms (i.e. acupuncture + concurrent therapy vs concurrent therapy) to minimize potential confounding by inclusion of concurrent therapies.

### Outcomes

Our primary outcomes were: (1) incidence of remission, defined according to individual study criteria; and (2) number of attacks per day after the completion of acupuncture therapy. Our secondary outcomes included: (1) incidence of a positive cold provocation test after the completion of acupuncture—this test involves immersing the affected digits into cold water and observing whether a Raynaud’s attack is triggered using photoplethysmography (a positive test is defined as a successfully triggered attack);^11^ and (2) incidence of a positive cold stimulation test after the completion of acupuncture—this test involves immersing the affected digits into cold water and measuring the length of time required for the digits to return to a normal temperature (a positive test is defined as the restoration of normal temperature before a predefined time point).^12^

### Study selection and data extraction

Two authors performed title and abstract screening in duplicate based on the aforementioned eligibility criteria. Abstracts deemed to be relevant were then entered into a duplicate full-text screening process. We resolved disagreements by consulting with a senior author (J.D.) to reach consensus.

We carried out data extraction in duplicate using prospectively developed data extraction sheets. Disagreements were resolved by consulting the senior author (J.D.) to review the data. For studies with missing information, we made attempts to contact the corresponding and/or first authors of these studies to obtain unpublished data.

### Risk of bias

We evaluated the risk of bias of included studies in duplicate using the Cochrane Collaboration’s revised tool for assessing risk of bias (RoB) in randomized trials (RoB 2).^13^ RoB for included studies was rated using the signaling questions and algorithm maps provided by the RoB 2 guidance document.^14^

### Meta-analysis

We conducted all statistical analyses using R 4.0.0, and we performed random effect meta-analyses using the meta 4.12-0 library. For the outcome of remission incidence and our secondary outcomes, we expressed and pooled treatment effects as risk ratios (RRs) with corresponding 95% confidence intervals (CIs). We also calculated the number needed to treat (NNT) for these outcomes. For continuous outcomes, that is, the number of attacks per day, we expressed and pooled the treatment effect as weighted mean differences (WMDs) with corresponding 95% CIs.

### Heterogeneity assessment

We assessed the presence of heterogeneity using Cochran’s Q test, and we considered a p_Q_ < 0.10 as statistically significant.^15^ We then quantified heterogeneity using I^2^ statistics.^15^ I^2^ values were interpreted based on recommendations from the Cochrane Handbook.^15^

### Meta-regression

We performed meta-regression analyses on several study-level covariates to explore sources of heterogeneity. Our covariates of interest included: (1) mean age; (2) gender (percentage of female patients); (3) primary/secondary Raynaud’s syndrome (percentage of patients with primary Raynaud’s syndrome); and (4) length of follow-up.

### Influence analysis

We identified outliers with extreme treatment effects within each meta-analysis using influence analyses and Graphical display Of Study Heterogeneity (GOSH) analyses.^16,17^ If we detected outliers, we performed sensitivity analyses excluding the outlying studies to examine their effects on the pooled effect size and heterogeneity measures.

### Publication bias

We drew funnel plots and used Egger’s regression test to identify publication bias within the included studies.^18^ We also visually inspected the funnel plots for signs of asymmetry, as the Egger’s test lacks power to detect publication bias when there are fewer than 10 studies.^15^

### Quality of evidence

Quality of evidence was assessed using the Grading of Recommendations, Assessment, Development and Evaluations (GRADE) framework^19^ for each outcome.

### Network meta-analysis

For the outcome of remission incidence and number of daily attacks, we encountered several different forms of acupuncture, specifically manual acupuncture (MA) with moxibustion and electroacupuncture (EA). As these procedures differ from standalone MA, it was considered that they may result in different treatment effects. To compare the effects of different forms of acupuncture procedure, we conducted random effects network meta-analyses (NMAs) using gemtc 0.8-4. Treatment effects in terms of remission incidence were expressed as RRs with 95% credible intervals (CrIs), and treatment effects in terms of the number of daily attacks were expressed as WMDs with 95% CrIs. We ranked the treatments in terms of their efficacy using surface under the cumulative ranking curve (SUCRA) scores.^20^ The heterogeneity associated with the NMAs was examined using I^2^ statistics. The quality of evidence within each network was assessed using the Confidence in Network Meta-Analysis (CINeMA) application,^21^ based on the GRADE framework.

## Results

### Study selection

**Figure 1.**
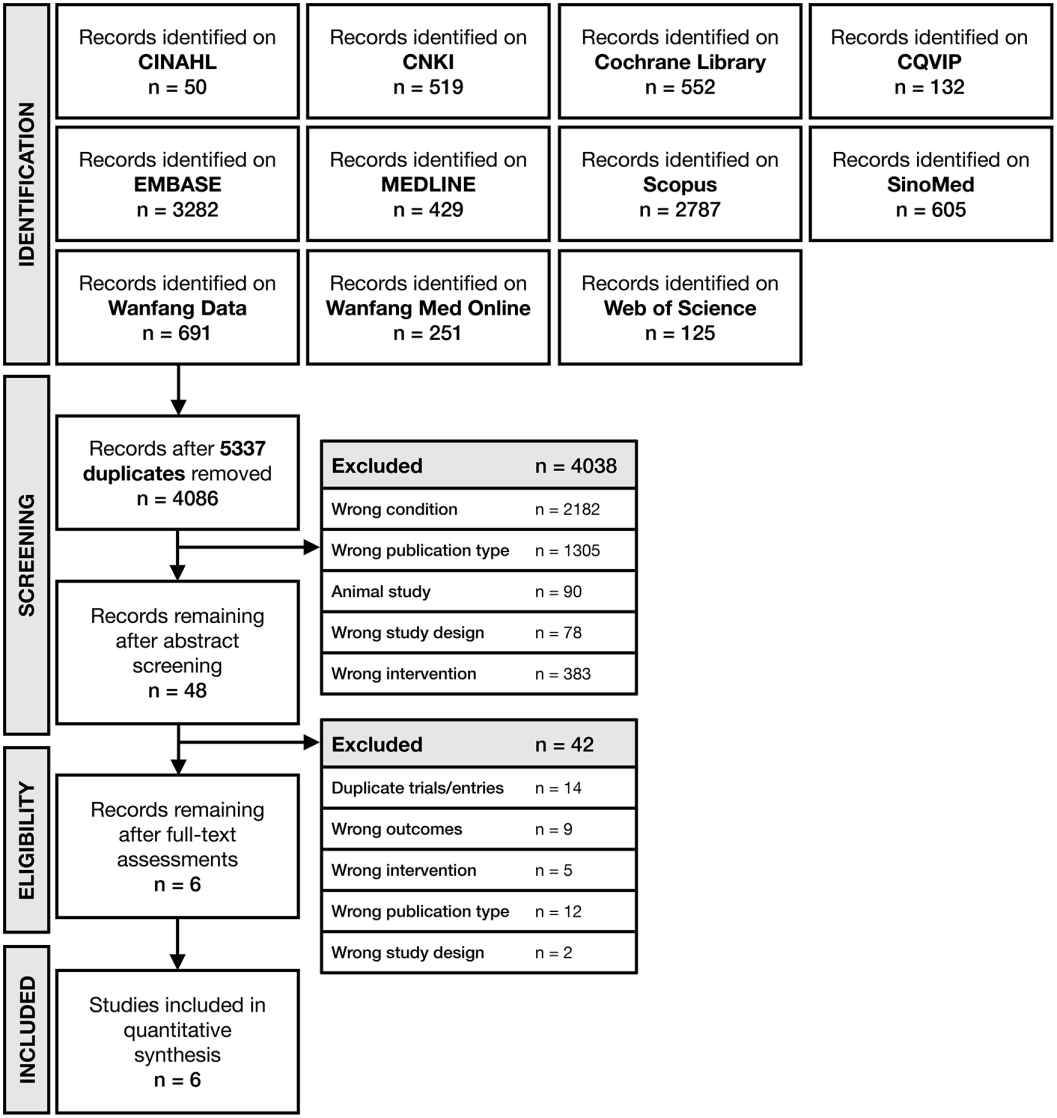
PRISMA flowchart for the identification and selection of randomized controlled trials. CINAHL: Cumulative Index of Nursing and Allied Health Literature; CNKI: China National Knowledge Infrastructure; CQVIP: Chongqing VIP Information.

Our study selection and screening process is shown in Figure 1. In total, we retrieved 4086 abstract entries from our database search; only 48 abstracts were included in the full-text screening. We excluded 42 full-text studies that were duplicate trials, had ineligible comparisons with the use of different concurrent therapies between treatment arms, did not report any of our outcomes of interest, or represented observational studies, reviews, editorials or other irrelevant publications.

### Study characteristics

**Figure 2.**
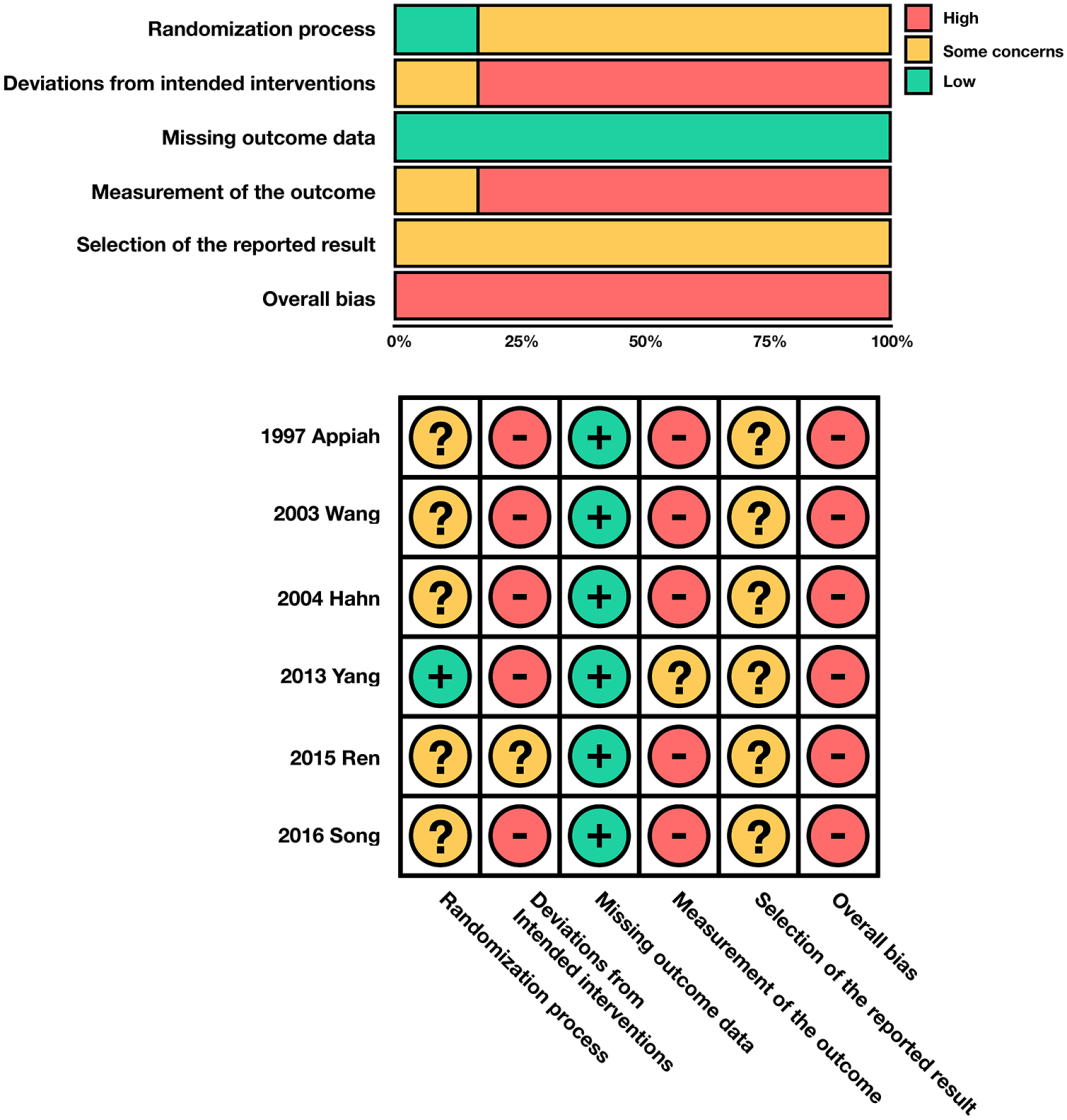
Risk of bias 2.0 ratings for included studies.

We included six RCTs published between 1997 and 2016 with 272 patients in our meta-analysis (see Table 1).^8,22–26^ Two studies (33%) used verum/traditional MA,^8,25^ two studies (33%) used MA + moxibustion,^22,23^ and two studies (33%) used EA.^24,26^ Four studies (66%) included patients with primary Raynaud’s syndrome,^22,24–26^ one study (17%) included patients with Raynaud’s syndrome secondary to systemic sclerosis, mixed connective tissue disease or systemic lupus erythematosus,^8^ and one study did not report the classification of Raynaud’s syndrome that was included.^23^ The number of acupuncture sessions varied from 7 to 50, and the follow-up period varied from 15 to 91 days. All studies were deemed to be at high risk of overall bias, mainly due to the absence of prospectively registered study protocols, unreported and/or infeasible blinding of participants, healthcare providers and outcome assessors including lack of sham interventions (Figure 2).

**Table 1.** Characteristics of included trials.

Study	Treatment	Concurrent therapy	Sample size	Age (mean ± SD or range)	Sex (M/F)	Primary Raynaud’s syndrome (%)	Mean disease duration, months (mean ± SD or range)	Follow-up (days)	Number of acupuncture treatments
Appiah et al.^22^	MA + moxibustion	None	16	41.5 ± 10.7	5/11	100	16.1 ± 14.6	91	7, once every other day over 2 weeks
	No treatment		17	45.5 ± 11.5	5/12	100	11.4 ± 11.1	91	–
Wang et al.^23^	MA + moxibustion	Metoprolol 100 mg/day	30	26–58	9/21	–	3–36	15	15, once per day
	No treatment		30	24–57	7/23	–	2–34	15	–
Hahn et al.^8^	MA	None	11	47 ± 12	1/10	0	–	56	8, once per week
	Off-point sham acupuncture		8	41 ± 11	2/6	0	–	56	–
Yang et al.^11^	EA	Herbal medicine (safflower injection), cyclophosphamide, buflomedil	30	37.70 ± 8.22	0/30	100	4.17 ± 1.26	30	30, once per day
	No treatment		30	37.97 ± 8.71	0/30	100	4.57 ± 1.77	30	–
Ren and Yu^25^	MA	Reserpine 1 mg/day	30	58	11/19	100	1–60	60	50, once per day with 3-day rest after every 10 treatments
	No treatment		30	57	13/17	100	1–60	60	–
Song et al.^26^	EA	Herbal medicine (safflower yellow injection), alprostadil	20	33	10/50	100	2–24	30	30, once per day with 5-day rest after every 15 treatments
	No treatment		20			100		30	–

SD: standard deviation; M: male; F: female; MA: manual acupuncture; EA: electroacupuncture.

### Remission incidence

**Figure 3.**
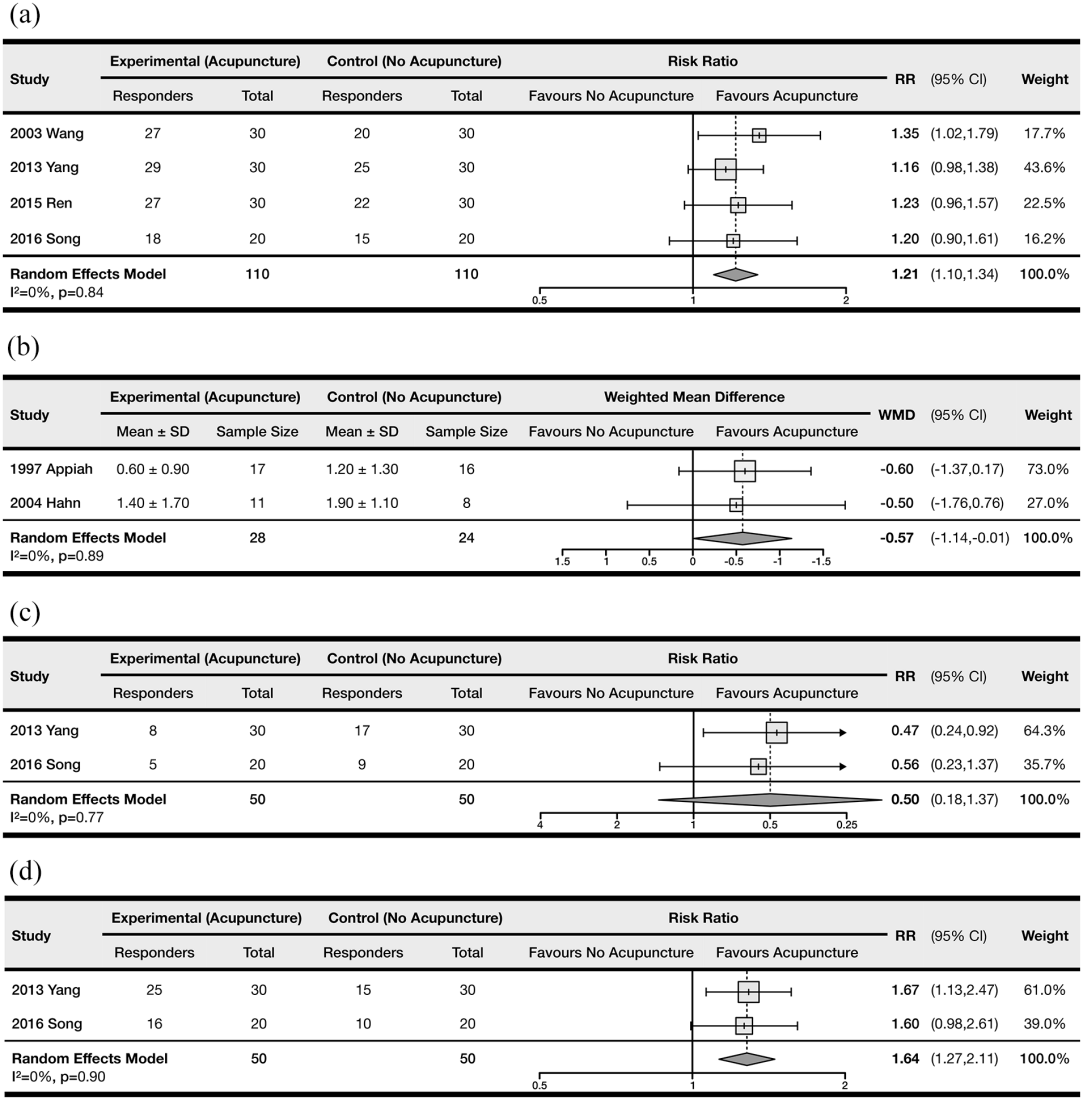
Forest plot comparing the treatment efficacy between control and acupuncture groups: (a) remission incidence, (b) number of daily attacks, (c) incidence of positive cold provocation test, and (d) incidence of positive cold stimulation test. RR: relative risk; MD: mean difference; CI: confidence interval.

Figure 3(a) shows the meta-analysis forest plot for remission incidence. Four RCTs (n = 220 patients) were included in this analysis. A majority of the included studies (three studies, 75%) defined remission as a reduction in severity of symptoms, increase in cold endurance of the digits, and improvements as observed by nailfold capillaroscopy.^23,24,26^ One study (25%) defined remission as a reduction in the severity of symptoms only.^25^

The use of acupuncture was associated with a statistically significant increase in the incidence of remission (RR = 1.21, 95% CI = 1.10 to 1.34; NNT = 6.39). There was a lack of heterogeneity among the included studies (p_Q_ = 0.84, I^2^ = 0%). We did not identify any outliers using influence and GOSH analyses, nor did we detect the presence of small study effects using funnel plots (Egger’s test, p = 0.27; see Supplemental Figure S1). Meta-regression analyses of study-level covariates revealed no significant correlations between remission incidence and sex (p = 0.34), mean age (p = 0.47), or duration of follow-up (p = 0.74). We did not complete a meta-regression for primary/secondary Raynaud’s syndrome, as only studies with primary Raynaud’s syndrome included this outcome. Overall, the meta-analysis was based on low-quality evidence according to GRADE, due to high within-study risk of bias.

Because one study^25^ used a different definition of remission compared to the others, we examined the impact of excluding this study by conducting an influence analysis. We found that excluding this study did not impact the point estimate of the RR; however, the CI was expanded and crossed the line of no effect (RR = 1.21, 95% CI = 1.00 to 1.46). We suspect this could be due to the decrease in statistical power following the removal of the study, as there were no significant changes in heterogeneity following this study’s exclusion.

#### Network meta-analysis

**Figure 4.**
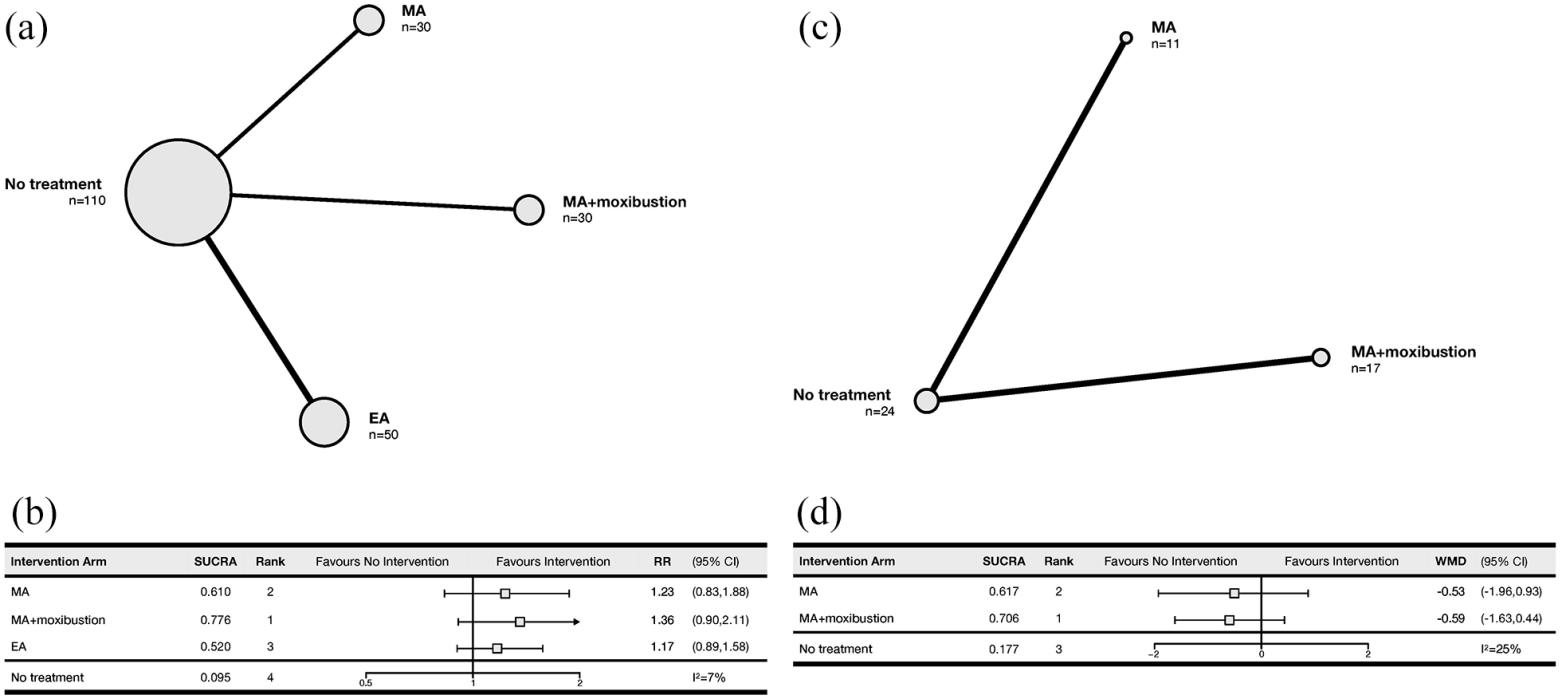
Network and forest plots for network meta-analyses. The size of the nodes represents the relative number of patients, and the thickness of the edge represents the relative number of studies comparing the connected treatment nodes in the network meta-analysis. (a) Network diagram for remission incidence. (b) Forest plot for remission incidence. (c) Network diagram for number of daily attacks. (d) Forest plot for number of daily attacks. MA: manual acupuncture; EA: electroacupuncture; SUCRA: surface under the cumulative ranking curve; RR: relative risk; MD: mean difference; CrI: credible interval.

Figure 4(a) and (b) show the network and forest diagrams, respectively, for the remission incidence NMA. MA + moxibustion (RR = 1.36, 95% CrI = 0.90 to 2.11), MA alone (RR = 1.23, 95% CrI = 0.83 to 1.88), and EA (RR = 1.17, 95% CrI = 0.89 to 1.58) were not associated with a significant increase in remission incidence compared with no treatment. According to SUCRA rankings, MA + moxibustion was likely to be the most efficacious in terms of increasing remission incidence (SUCRA 0.776), followed by MA alone (SUCRA 0.610), EA (SUCRA 0.520), and no treatment (SUCRA 0.095). The network experienced low heterogeneity (I^2^ = 7%). Overall, the NMA was based on very low-quality evidence due to high within-study risk of bias, imprecision and incoherence.

### Number of daily attacks

Figure 3(b) shows the meta-analysis forest plot for the number of daily attacks. Two RCTs (n = 52 patients) were included in the analysis.^8,22^ The use of acupuncture was associated with a statistically significant decrease in the number of daily attacks (WMD = −0.57, 95% CI = −1.14 to −0.01). There was a lack of heterogeneity among the included studies (p_Q_ = 0.89, I^2^ = 0%). We were unable to conduct a meta-regression, outlier analysis or Egger’s regression test due to the low number of included trials reporting this outcome. Overall, the meta-analysis was based on very low-quality evidence due to high within-study risk of bias and inability to examine publication bias.

#### Network meta-analysis

Figure 4(c) and (d) show the network and forest diagrams, respectively, for the number of daily attacks NMA. Both MA + moxibustion (WMD = −0.59, 95% CrI = −1.63 to 0.44) and MA alone (WMD = −0.59, 95% CrI = −1.63 to 0.44) were not significantly better than no treatment. According to SUCRA rankings, MA + moxibustion (SUCRA 0.706) was most likely to be the most efficacious in terms of reducing the number of daily attacks, followed by MA alone (SUCRA 0.617) and no treatment (SUCRA 0.177). The network experienced low heterogeneity (I^2^ = 25%). Overall, the NMA was based on very low-quality evidence due to high within-study risk of bias and concerns regarding imprecision, publication bias and incoherence.

### Cold provocation test

Figure 3(c) shows the meta-analysis forest plot for the incidence of positive cold provocation tests. Two RCTs (n = 100 patients) were included in the analysis.^24,26^ There was no significant effect of acupuncture on the incidence of positive results, based on our pooled treatment effect (RR = 0.50, 95% CI = 0.18 to 1.37; NNT = 3.85). There was a lack of heterogeneity among the included studies (p_Q_ = 0.77, I^2^ = 0%). We were unable to conduct a meta-regression, outlier analysis or Egger’s regression test due to the low number of included trials. The meta-analysis was based on very low-quality evidence due to high within-study risk of bias, imprecision and inability to examine publication bias.

### Cold stimulation test

Figure 3(d) shows the meta-analysis forest plot for incidences of positive cold stimulation tests. Two RCTs (n = 100 patients) were included in the analysis.^24,26^ The use of acupuncture was associated with a significant increase in the incidence of positive tests (RR = 1.64, 95% CI = 1.27 to 2.11; NNT = 3.13). There was a lack of heterogeneity among the included studies (p_Q_ = 0.90, I^2^ = 0%). We were unable to conduct a meta-regression, outlier analysis or Egger’s regression test due to the low number of included trials. The meta-analysis was based on very low-quality evidence due to high within-study risk of bias and inability to examine publication bias.

## Discussion

In this systematic review and meta-analysis, we investigated the use of acupuncture versus control in the treatment of Raynaud’s syndrome. We found that the use of acupuncture was associated with increased remission incidence, a decreased number of daily attacks, and an increased number of positive cold stimulation tests (which indicates an increased rate of temperature recovery in the digits). According to our results, acupuncture did not significantly decrease the incidence of attacks after cold provocation.

In addition, we conducted two NMAs for the outcome of remission incidence and number of daily attacks, respectively. Our findings suggested that none of the acupuncture treatments showed statistically significantly effectiveness compared with no treatment. However, our NMAs included a limited number of studies and our pooled estimates had wide 95% CIs. This indicates that, rather than being due to a lack of effect from acupuncture, our result may be more likely a consequence of insufficient statistical power, since our pairwise meta-analyses yielded promising results with little to no heterogeneity.

While the results from our pairwise meta-analyses support the use of acupuncture in patients with Raynaud’s syndrome, it must be noted that these findings were based on six studies with low sample sizes and high RoB. In addition, the GRADE and CINeMA rating showed that the quality of evidence provided by our meta-analyses and network meta-analyses was very low. These significant limitations were due to an overall lack of acupuncture RCTs involving Raynaud’s patients, as well as shortcomings commonly associated with Chinese RCTs, which often contain poor descriptions of their methodologies due to a lack of Chinese RCT reporting guidelines.^27^ Thus, we are only able to offer a weak recommendation for the use of acupuncture in patients with Raynaud’s syndrome, due to the high uncertainties associated with our findings.

Previous studies have shown that acupuncture can increase the blood flow volume in peripheral arteries by stimulating the autonomic nervous system,^28^ as well as improve local microcirculation^29^ and regulate the concentration of vasodilators such as nitric oxide.^30^ Acupuncture can also induce anti-nociceptive effects by increasing the release of adenosine; this property may help to relieve pain in patients with Raynaud’s syndrome.^31^ Because of these benefits of acupuncture, a previous systematic review has highlighted that acupuncture may be a viable alternative to conventional pharmacotherapies for treating Raynaud’s syndrome.^32^ The results of our meta-analysis lend limited support to this viewpoint; however, further investigations included larger and higher quality RCTs are needed to reinforce our findings.

### Conclusion

To our knowledge, this systematic review is the first knowledge synthesis study to investigate the use of acupuncture in patients with Raynaud’s syndrome. Our results suggest that acupuncture may be effective for Raynaud’s patients in terms of increasing remission incidence, decreasing number of daily attacks and increasing number of positive cold stimulation tests. However, due to the low sample sizes of the included RCTs, very low quality of evidence and high RoB, our findings should be taken with caution and validated using larger, higher quality RCTs.

## Supplemental Material

sj-docx-1-aim-10.1177_09645284221076504 – Supplemental material for The use of acupuncture in patients with Raynaud’s syndrome: a systematic review and meta-analysis of randomized controlled trials Click here for additional data file. Supplemental material, sj-docx-1-aim-10.1177_09645284221076504 for The use of acupuncture in patients with Raynaud’s syndrome: a systematic review and meta-analysis of randomized controlled trials by Fangwen Zhou, Emma Huang, Elena Zheng and Jiawen Deng in Acupuncture in Medicine
